# Multiple chikungunya virus introductions in Lao PDR from 2014 to 2020

**DOI:** 10.1371/journal.pone.0271439

**Published:** 2022-07-15

**Authors:** Elodie Calvez, Phaithong Bounmany, Somphavanh Somlor, Thonglakhone Xaybounsou, Souksakhone Viengphouthong, Sitsana Keosenhom, Paul T. Brey, Vincent Lacoste, Marc Grandadam

**Affiliations:** 1 Arbovirus and Emerging Viral Diseases Laboratory, Institut Pasteur du Laos, Vientiane, Lao People’s Democratic Republic; 2 Medical Entomology and Vector-Borne Disease Unit, Institut Pasteur du Laos, Vientiane, Lao People’s Democratic Republic; CEA, FRANCE

## Abstract

The first documented chikungunya virus (CHIKV) outbreak in Lao People’s Democratic Republic (Lao PDR) occurred in 2012–2013. Since then, several imported and a few autochthonous cases were identified by the national arbovirus surveillance network. The present study aimed to summarize the main genetic features of the CHIKV strains detected in Lao PDR between 2014 and 2020. Samples from Lao patients presenting symptoms compatible with a CHIKV infection were centralized in Vientiane Capital city for real-time RT-PCR screening. Molecular epidemiology was performed by sequencing the E2-6K-E1 region. From 2014 to 2020, two Asian lineage isolates (*e*.*g*. French Polynesia; Indonesia), one ECSA-IOL lineage isolate (*e*.*g*. Thailand) and one unclassified (*e*.*g*. Myanmar) were imported in Vientiane Capital city. Sequences from the autochthonous cases recorded in the Central and Southern parts of the country between July and September 2020 belonged to the ECSA-IOL lineage and clustered with CHIKV strains recently detected in neighboring countries. These results demonstrate the multiple CHIKV introductions in Lao PDR since 2014 and provide evidence for sporadic and time-limited circulation of CHIKV in the country. Even if the circulation of CHIKV seems to be geographically and temporally limited in Lao PDR, the development of international tourism and trade may cause future outbreaks of CHIKV in the country and at the regional level.

## Introduction

Chikungunya virus (CHIKV) is transmitted to humans by *Aedes* mosquito species [1]. With a single-stranded RNA positive sense genome, CHIKV belongs to the *Togaviridae* family, *Alphavirus* genus. During the acute phase of human infection, CHIKV infection is clinically characterized by sudden onset of high fever often accompanied by severe joint pain and other symptoms such as headache, maculopapular rash evolving to chronic poly arthralgia in up to one third of the patients [2]. Since 1952, CHIKV was recorded in more than 60 countries in Africa, Asia, Europe, the Southern Pacific region and the Americas [3–6]. The genetic polymorphism of the CHIKV genome allows to discriminate four lineages: the West African (WA), East/Central/South African (ECSA), ECSA-derived Indian Ocean (ECSA-IOL) and Asian [7]. In Southeast Asia, the circulation of the Asian lineage was described since the 1950s and an active co-circulation of the Asian, ECSA and ECSA-IOL lineages was highlighted since the beginning of the 2000’s [3, 5, 6].

Lao People’s Democratic Republic (Lao PDR) is a land-locked, least developed country located in the middle of the Indochinese peninsula that progressively opens its borders to international exchanges for more than a decade now. This central location increases the risk of arbovirus introductions as previously reported for dengue viruses (DENV) [8–13] and CHIKV [14, 15]. In 2012–2013, the first chikungunya outbreak was described in the Southern provinces of the country and phylogenetic studies revealed that the Lao strains belonged to the ECSA-IOL lineage with a Cambodian origin [14, 16]. Since then and until 2020, no outbreak of CHIKV has been recorded in the country. Here, we studied the phylogenic relationships of the few imported and autochthonous CHIKV cases identified between 2014 and 2020 in Lao PDR.

## Methods

### Ethic statement

Ethics approval was obtained from the Lao National Ethics Committee for Health Research of the Ministry of Health of Lao PDR (N°114/NECHR).

For the patient samples included in this study, participant or a parent or a legal guardian provided a written informed consent.

### Human samples collection and screening

Samples of clinically-suspected CHIKV cases were collected by hospitals belonging to the arbovirus surveillance network implemented in 2012 in Lao PDR and coordinated by the Institut Pasteur du Laos [8, 9, 14]. Viral genomic RNA was extracted from human plasmas or culture supernatants using a Nucleospin DX or Nucleospin 96 core kit purification kit according to the manufacturer’s instructions (Macherey-Nagel) and samples were screened using real-time RT-PCR assays [17].

Samples from autochthonous cases from Bolikhamxay have been tested for the presence of anti-CHIKV antibodies. Detection of IgM and IgG was performed using SD Biosensor STANDARD Q Arbo Panel I (Z/D/C/Y) kit (SD Biosensor) and an in-house ELISA method as described elsewhere, respectively [18, 19].

### Virus amplification

Our previous experience revealed that direct sequencing failed on samples (either plasmas or culture supernatants) displaying Ct values above 28 [14]. Therefore, virus isolation was attempted for samples with a low viral load, and for which a leftover of at least 150 μL of original plasma was available. Part of the remaining volume (between 100 and 200μL) was used to perform one or two passages on Vero E6 cells maintained in culture at 37°C with 5% CO_2_ in Medium 199 (Gibco™, Thermo Fisher Scientific, Waltham, MA, USA) supplemented with 2% fetal bovine serum (FBS; Gibco™, Thermo Fisher Scientific, Waltham, MA, USA). After five days of incubation, supernatants were collected and stored at -80°C before analysis.

### E2-6K-E1 gene amplification

A DNA fragment encompassing the E2-6K-E1 region (2,772 bp) was prepared from RNA purified from plasma or culture supernatant by RT-PCR using primers set described elsewhere [14, 20] (S1 Data). Amplification products were sequenced using primers used for the RT-PCR amplification as well as internal primers (S1 Data) [9]. Briefly, after generating the first stand cDNA using a Maxima H Minus First Stand cDNA Synthesis kit (Thermo Scientific, Waltham, MA, USA), Phusion Flash High-Fidelity PCR Master Kit (New England Biolabs^®^ Inc, Waltham, MA, USA) was used for the overlapping PCRs. Amplification products were then purified by ExoSAP-IT^TM^ PCR Product Cleanup Reagent (Thermo Fisher Scientific, Waltham, MA, USA). Sequencing was performed on both strands using BigDye Terminator v3.1 Cycle sequencing kit (Applied Biosystem, Waltham, MA, USA) on a Genetic Analyzer 3500xL (Applied Biosystem, Waltham, MA, USA).

### Phylogenetic analysis

Raw sequences were analyzed and edited in BioEdit version 7.0.5.3 software (Manchester, United Kingdom). Then, a multiple-sequence alignment was built using a panel of reference sequences retrieved from GenBank using the ClustalW program and the alignment was checked manually (S2 Data). The sequences were translated into amino acids, and both nucleotide and amino acid sequences were checked for irregularities. Pairwise sequence identity (at the nucleotide and amino acid levels) of the E2-6K-E1 coding sequences was calculated with MEGA version 7 (www.megasoftware.net) using uncorrected p-distances. A phylogenetic tree was inferred from the aligned nucleotide sequences by using a maximum likelihood phylogenetic approach [9, 14]. The best-fitted model of nucleotide substitution was determined using MEGA v7 under corrected Akaike information criteria (AICc). The Kimura-2 model was identified as the optimal model of nucleotide evolution. One thousand bootstrap replicates were generated.

## Results

### Epidemiological analysis of the patients infected with CHIKV

Suspected CHIKV cases, matching with a case definition of CHIKV infection, were identified in Lao PDR between 2014 and 2020, corresponding to either imported cases or to autochthonous cases in Bolikhamsay and Savannakhet provinces.

**Fig 1:**
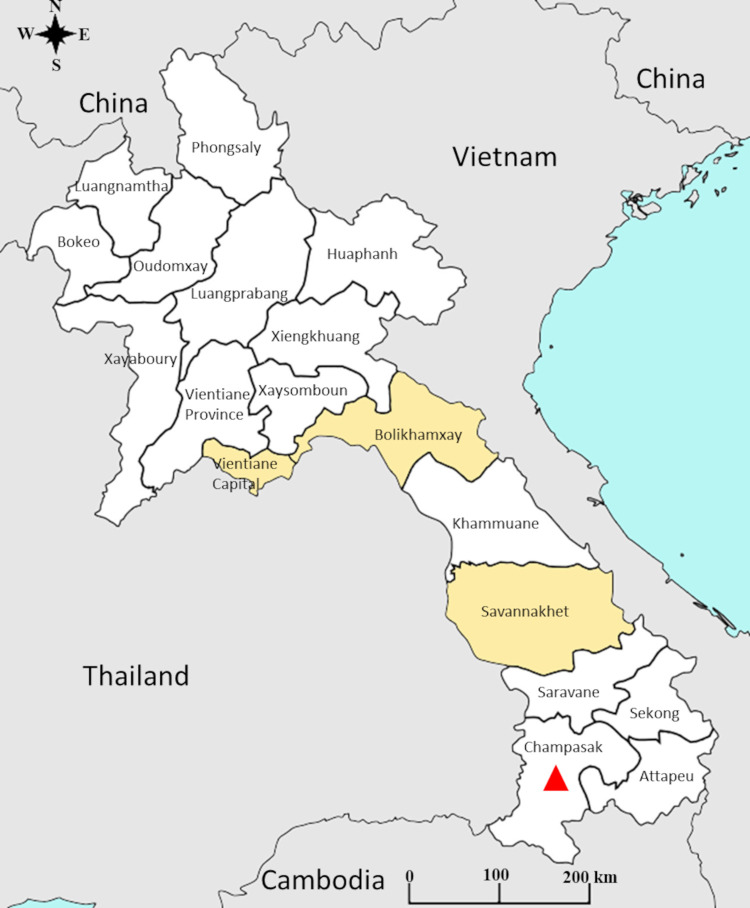
Geographical location of chikungunya cases, at the province level, in Lao PDR. Data based on the analysis of the samples collected by the Institut Pasteur du Laos arbovirus surveillance network between 2012 and 2020. The provinces indicated in yellow correspond to those where at least one sample was found positive for chikungunya virus during the study period (2014–2020). The red triangle indicates the province where the chikungunya outbreak occurred in 2012–2013.

A total of 18 cases were confirmed in Lao PDR between 2014 and 2020 of which four were imported cases from French Polynesia, Indonesia, Myanmar and Thailand, respectively (S3 Data). All autochthonous cases were recorded between July and September 2020 in one district of the central province of Bolikhamxay and in another one of the Southern province of Savannakhet (Fig 1). Among the samples collected in Bolikhamxay (N = 32), four were positive for anti-CHIKV IgM only, ten for IgM/IgG and 11 for IgG. These 25 anti-CHIKV antibody positive samples include four of the eight confirmed cases by RT-PCR from Bolikhamxay. Given the low volume (less than 250μL) of the samples from Savannakhet and from the imported cases, molecular tests have been prioritized.

In Bolikhamxay, eight CHIKV positive samples were detected. The mean age of the positive cases was 23.25 years (range 10–39 years). Among them, seven samples were from men and one from a woman. Of the six confirmed cases from Savannakhet, three were men and the other three women. The mean age for the Savannakhet’ patients was 60.33 years (range 30–68 years).

### Genetic diversity of sequences

In this series, E2-6K-E1 sequences were obtained from six samples from either plasmas or culture supernatants. These six samples had a low Ct value ≤ 25 (Table 1). We were unable to obtain the sequence from the imported case from Myanmar due to the low viral load (Ct = 38) and virus isolation attempts were unsuccessful.

**Table 1:** Data relative to the Lao CHIKV strains included in the phylogenetic analysis.

Sample Identification	Date of collection	Location	Patient age (years)	Patient gender	Patient symptoms	Biological sample	Genbank accession Number
CV001	November 2014	Vientiane Capital (ex French Polynesia)	53	Male	fever, arthralgia, rash	Culture supernatant *	MZ292728
19–9293	February 2019	Vientiane Capital (ex Indonesia)	44	Male	fever, headache	Culture supernatant *	MZ292729
20–16862	August 2020	Vientiane Capital (ex Thailand)	36	Male	fever, headache, myalgia, retro-orbital pain, nausea, rash	Culture supernatant *	MZ292733
20–16657	July 2020	Savannakhet Province	65	Male	fever, arthralgia, myalgia, headache	Plasma	MZ292732
Bolikhamxay-14	July 2020	Bolikhamxay Province	39	Male	fever, arthralgia, myalgia	Plasma	MZ292730
Bolikhamxay-30	July 2020	Bolikhamxay Province	19	Male	fever, arthralgia, retro-orbital pain	Plasma	MZ292731

* One or two passages were performed on Vero E6 cells.

The six strains exhibited between themselves from 92.6 to 99.9% nucleotide identity each other and from 96.1 to 99.9% amino acid identity over the 2,772 bp / 923 aa sequences. The three sequences from the 2020 autochthonous cases as well as the one from the imported case from Thailand displayed from 99.7 to 99.9% nucleotide identity and from 99.6 to 99.9% amino acid identity. They exhibited from 99.6 to 100% nucleotide identity and from 99.7 to 100% amino acid identity with CHIKV strains identified in different Asian countries (Thailand, Myanmar, China, Bangladesh and Malaysia) since 2017. They also showed respectively from 98.4 to 98.7% and from 98.8 to 99.1% nucleotide and amino acid identity with the 2012–2013 Lao strains from Champassak. In addition, the sequence from the imported case from French Polynesia was identical to the one (GenBank accession number KR559473) described in 2015. At last, the sequence from the case imported from Indonesia exhibited from 99.2 to 99.7% nucleotide identity and from 99.6 to 99.8% amino acid identity with sequences from Indonesia described from 2013 to 2018.

Looking at specific amino acid signatures, the three autochthonous sequences here described from Bolikhamxay and Savannakhet and the four ones identified in Champassak in 2013 showed a cumulative number of 30 amino acid substitutions over 923 positions as compared to the reference S27 strain (NP_690589.2): 20 common to all strains, seven specific to either the 2013 or the 2020 strains, one (F84S in E2) shared between the two sequences from Bolikhamxay with one of the three sequences from Champassak (LN901352), and two (S60N in 6K and A359G in E1) restricted to a single strain (LN901359 and MZ292731, respectively). Twenty polymorphic positions were located in E2 (G57K, I74M, G79E, F84S, T110S, N160T, A164T, L181M, S194G, G205S, I211T, K252Q, V264A, M267R, N273I, S299N, T312M, A344T, S375T and V386A), three in 6K (V8I, I54V and S60N) and eight in E1 (K211E, A226V, M269V, D284E, I317V, V322A and A359G). Focusing on the main genetic signatures for the E1 and E2 envelope glycoproteins, K211E and I317V in E1 were specific to the 2020 strains while A226V was specific to the 2013 strains. Regarding E2, T110S and K252Q were restricted to the 2013 strains while G205S and V264A were specific to the 2020 strains.

### Phylogenetic analysis of CHIKV

**Fig 2:**
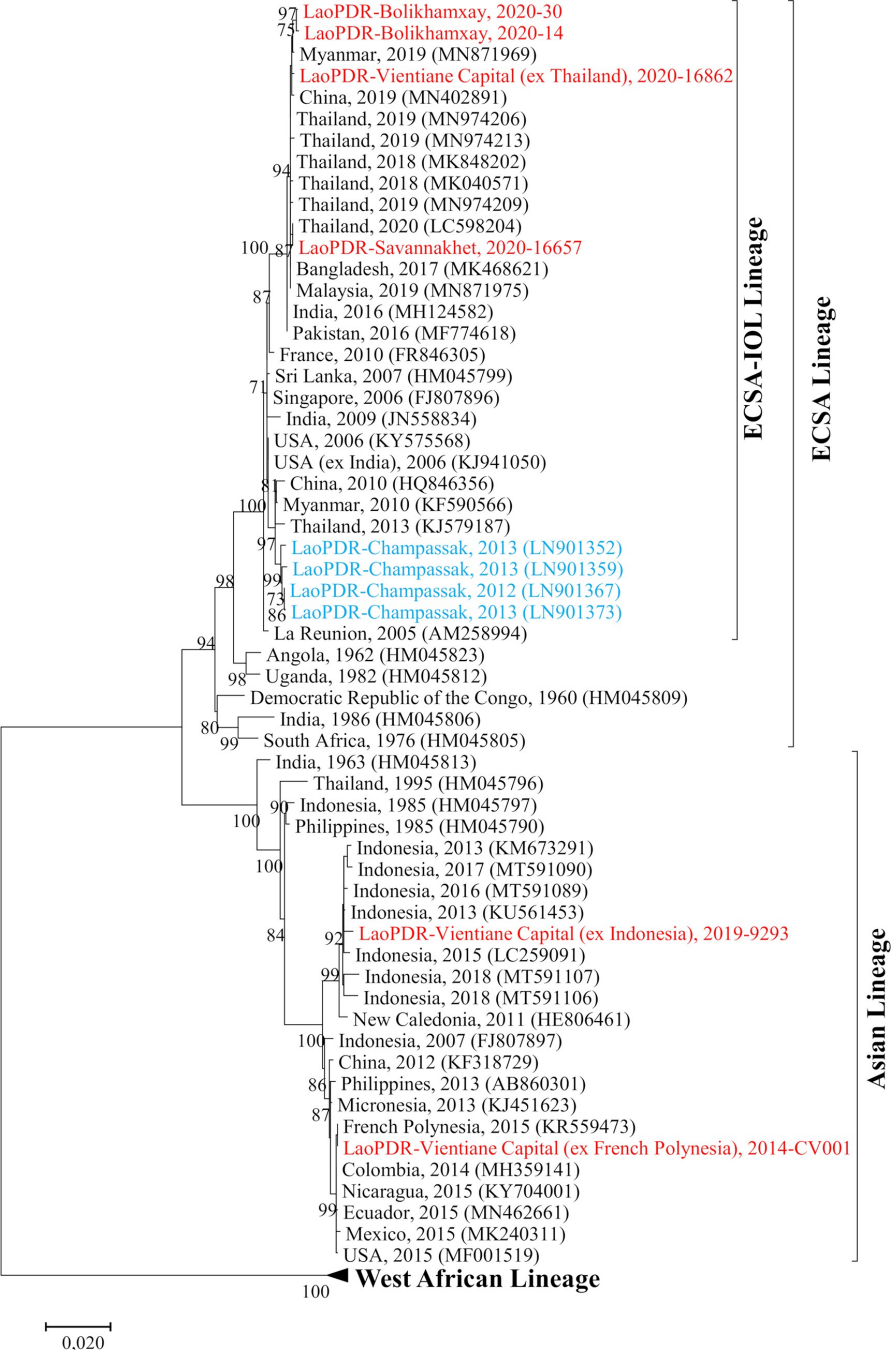
Maximum-likelihood phylogenetic tree of CHIKV sequences from Lao PDR based on E2-6K-E1 region. Only bootstrap values >70 are shown. The Lao strains sequenced in this study are indicated in red, the previously identified ones in blue.

On a phylogenetic viewpoint, the imported strains from French Polynesia and Indonesia belonged to the Asian lineage whereas the one imported from Thailand belonged to the ECSA-IOL lineage (Fig 2). Within the Asian lineage, the strain from Indonesia grouped into a cluster of sequences from Indonesia identified between 2013 and 2018 while the one imported from French Polynesia was associated with another strain from French Polynesia (KR559473) within a cluster of sequences from the Americas. In addition, the 2020 autochthonous Lao strains as well as the one imported from Thailand belonged to the ECSA-IOL lineage. They all fell within a cluster of Asian sequences from Thailand, China, Myanmar, Bangladesh and Malaysia identified between 2017 and 2020. Within the ECSA-IOL lineage, these strains grouped in a cluster independent from the former one gathering the Champassak 2013 isolates (Fig 2).

## Discussion

The circulation of CHIKV has been poorly documented in Lao PDR since the 2012–2013 outbreak [14, 15]. As CHIKV actively circulates around the world and in neighboring countries, such as in China, Thailand and Myanmar [21–24], this creates a threat for CHIKV re-introduction and spread in Lao PDR. Since 2014, the geographic diversity of the imported cases (French Polynesia; Indonesia; Thailand; Myanmar) and the occurrence of local transmissions support this assumption.

As for the 2012–2013 outbreak [14–16], the demographic profile of the patients seemed to demonstrate an active circulation of CHIKV among the general population in Lao PDR in 2020. However, the virus circulation appeared to be geographically and temporally limited. After the 2012–2013 outbreak in Champassak province, seroprevalence study revealed a medium to high IgM/IgG seroprevalence levels ranging from 33% to 94% in selected villages in the province [14]. The population in Lao PDR is young (half of the population is under 24 years old) (www.lsb.gov.la). Since the 1960s, only few studies investigated the presence of anti-CHIKV antibodies among the Lao population but in restricted areas, Champassak province and Vientiane capital [14, 25, 26]. In absence of a seroprevalence study at the country level this suggests a low immunity against CHIKV among the Lao population that could promote the introduction and emergence of CHIKV in the country.

The CHIKV sequences identified in 2020 in Bolikhamxay and Savannakhet provinces belonged to the ECSA-IOL lineage. Interestingly, they grouped into a different cluster independent from the ones identified during the 2012–2013 outbreak (98.6% of nucleotide identity) [14]. In addition, they clustered with recent sequences isolated in Thailand between 2019 and 2020 as well as in neighboring countries such as Myanmar, where CHIKV outbreaks were recorded, or China where imported cases from Myanmar and Thailand were detected [22, 27–29].

At the amino acid level, CHIKV sequences from autochthonous Lao cases identified in 2020 harbored common substitutions with those of 2012–13. However, others substitutions are specific to the 2012–13 strains from Champassak or to the 2020 strains from Bolikhamxay and Savannakhet. Three positions, one in E1 (A226V) and two in E2 (T110S and K252Q) were restricted to the 2012–13 strains while four others, two in E1 (K211E and I317V) and the other two (G205S and V264A) in E2, were specific to the 2020 strains. These specific substitutions, as well as the phylogenetic closeness of the 2020 autochthonous Lao strains with others identified in neighboring countries at the same period are in support of an independent reintroduction of the virus in 2020 rather than their evolution from the 2012–13 strains. These genetic links are thus in favor of an active circulation of the virus at a regional level. It is noteworthy that substitutions within the CHIKV genome can have an impact on its transmissibility by the vectors [20, 30, 31]. Indeed, as it has previously been demonstrated, the presence of a single mutation in the E1 and E2 region could influence the virus transmission by *Aedes* [32–37]. Further investigations on the impact of the mutations here described on virus transmission should be carried out with the main *Aedes* vectors in Lao PDR, *Ae*. *aegypti* and *Ae*. *albopictus* [9, 14, 38, 39].

Overall, the results obtained in this study highlight the re-introduction and a limited circulation of novel CHIKV strains in Lao PDR. Indeed, even if CHIKV sequences obtained in 2012–2013 and 2020 are closely related (98.4–98.7% of nucleotide identity) between each other, the presence of specific mutations within E2-6K-E1 region is correlated with the circulation of new variants in the country as already observed in several countries in Southeast Asia especially in Thailand [23, 28] and in China [40]. Furthermore, as described in this study, phylogenetic studies on CHIKV and DENV in Lao PDR demonstrated that introduction and spread of arboviruses in this country are correlated with their circulation at the regional level, particularly in countries neighboring such as Thailand, Cambodia, Myanmar and China [8–10, 12, 14]. The central geographic position of Lao PDR in Southeast Asia could promote this risk of arbovirus introduction from neighboring countries due to human mobility [41–43] and should be further investigated due to development of the international trade through, for example, the Lao-China rail connectivity.

Up until now, only the ECSA-IOL lineage has been involved in the autochthonous circulation of CHIKV in Lao PDR, which is intriguingly limited to the Central and Southern regions of the country while the imported cases were detected in Vientiane Capital. However, several factors associated with the virus (genetic, viremia), its vector(s) (density, lifespan, vector competence), the host (immunity), and the environment (temperature, humidity) can influence the transmission of arboviruses. These factors and their interactions have to be investigated to prevent and control future CHIKV outbreaks in Lao PDR.

## Supporting information

S1 Data List and position of primers used for RT-PCR and sequencing of the E2-6K-E1 region. (DOCX)

S2 Data References of CHIKV E2-6K-E1 sequences from GenBank used in this study. (DOCX)

S3 Data Information relative to the Lao chikungunya samples collected between 2014 and 2020 and found positive by RT-PCR. (DOCX)
