# Supplementary material for: Multiple chikungunya virus introductions in Lao PDR from 2014 to 2020
Source: PLoS One. 2022 Jul 15;17(7):e0271439. doi: 10.1371/journal.pone.0271439 (PMC9286254; doi:10.1371/journal.pone.0271439)
Supplement: S1 Data — (DOCX) [file pone.0271439.s001.docx]

**Supplementary data 1.** List and position of primers used for RT-PCR and sequencing of the E2-6K-E1 region.

| **Fragment** | **Forward** | **Reverse** | **Publication** |
| --- | --- | --- | --- |
| FG17 | TGCTTGAGGACAACGTCATGAG | TTTGTGATTGGTGACCGCG | Schuffenecker I, *et al*, 2006 |
| FG18 | AGTCCGGCAACGTAAAGATCAC | AAAGGTTGCTGCTCGTTCCAC |  |
| FG19 | AGTTGTGTCAGTGGCCTCGTTC | TAAAGGACGCGGAGCTTAGCTG |  |
| FG20^a^ | ACAAAACCGTCATCCCGTCTC | TGACTATGTGGTCCTTCGGAGG |  |
| FG21^a^ | CAGCAAGAAAGGCAAGTGTGC | TTTGCCAATTATGGTATTCA |  |
| FGT5^b^ | GTGGAGAAGTCCGAATCA | TTAAAGAKGGCGCGTCGA | This study |
| FGT6^b^ | AGCAACAAACCCGGTAAG | GTGTGTCTCTTSRGGGAC |  |

^a^ indicates the primers used for the ECSA-IOL lineage sequencing.

^b^ indicates the primers used for the Asian lineage sequencing.
