# Supplementary material for: Multiple chikungunya virus introductions in Lao PDR from 2014 to 2020
Source: PLoS One. 2022 Jul 15;17(7):e0271439. doi: 10.1371/journal.pone.0271439 (PMC9286254; doi:10.1371/journal.pone.0271439)
Supplement: S2 Data — (DOCX) [file pone.0271439.s002.docx]

**Supplementary data 2.** References of CHIKV E2-6K-E1 sequences from GenBank used in this study.

| **Country** | **Year of collection** | **GenBank accession number** | **CHIKV lineage** |
| --- | --- | --- | --- |
| Angola | 1962 | HM045823 | ECSA |
| Bangladesh | 2017 | MK468621 | ECSA-IOL |
| China | 2012 | KF318729 | Asian |
| China | 2019 | MN402891 | ECSA-IOL |
| China | 2010 | HQ846356 | ECSA-IOL |
| Colombia | 2014 | MH359141 | Asian |
| Democratic Republic of the Congo | 1960 | HM045809 | ECSA |
| Ecuador | 2015 | MN462661 | Asian |
| France | 2010 | FR846305 | ECSA-IOL |
| French Polynesia | 2015 | KR559473 | Asian |
| India | 2016 | MH124582 | ECSA-IOL |
| India | 1963 | HM045813 | Asian |
| India | 2009 | JN558834 | ECSA-IOL |
| India | 1986 | HM045806 | ECSA |
| Indonesia | 2016 | MT591089 | Asian |
| Indonesia | 2007 | FJ807897 | Asian |
| Indonesia | 2013 | KM673291 | Asian |
| Indonesia | 2018 | MT591107 | Asian |
| Indonesia | 2018 | MT591106 | Asian |
| Indonesia | 2017 | MT591090 | Asian |
| Indonesia | 1985 | HM045797 | Asian |
| Indonesia | 2013 | KU561453 | Asian |
| Indonesia | 2015 | LC259091 | Asian |
| Ivory Coast | 1993 | HM045820 | West African |
| Ivory Coast | 1993 | HM045818 | West African |
| La reunion | 2005 | AM258994 | ECSA-IOL |
| Lao PDR-Champassak | 2013 | LN901359 | ECSA-IOL |
| Lao PDR-Champassak | 2012 | LN901367 | ECSA-IOL |
| Lao PDR-Champassak | 2013 | LN901352 | ECSA-IOL |
| Lao PDR-Champassak | 2013 | LN901373 | ECSA-IOL |
| Malaysia | 2019 | MN871975 | ECSA-IOL |
| Mexico | 2015 | MK240311 | Asian |
| Micronesia | 2013 | KJ451623 | Asian |
| Myanmar | 2019 | MN871969 | ECSA-IOL |
| Myanmar | 2010 | KF590566 | ECSA-IOL |
| New Caledonia | 2011 | HE806461 | Asian |
| Nicaragua | 2015 | KY704001 | Asian |
| Nigeria | 1965 | HM045807 | West African |
| Nigeria | 1964 | HM045786 | West African |
| Pakistan | 2016 | MF774618 | ECSA-IOL |
| Philippines | 2013 | AB860301 | Asian |
| Philippines | 1985 | HM045790 | Asian |
| Senegal | 1966 | HM045816 | West African |
| Senegal | 1983 | AY726732 | West African |
| Senegal | 2005 | HM045817 | West African |
| Senegal | 1993 | HM045819 | West African |
| Singapore | 2006 | FJ807896 | ECSA-IOL |
| South Africa | 1976 | HM045805 | ECSA |
| Sri Lanka | 2007 | HM045799 | ECSA-IOL |
| Thailand | 2018 | MK040571 | ECSA-IOL |
| Thailand | 2019 | MN974213 | ECSA-IOL |
| Thailand | 2019 | MN974209 | ECSA-IOL |
| Thailand | 2018 | MK848202 | ECSA-IOL |
| Thailand | 1995 | HM045796 | Asian |
| Thailand | 2013 | KJ579187 | ECSA-IOL |
| Thailand | 2019 | MN974206 | ECSA-IOL |
| Thailand | 2020 | LC598204 | ECSA-IOL |
| Uganda | 1982 | HM045812 | ECSA |
| USA | 2006 | KY575568 | ECSA-IOL |
| USA | 2015 | MF001519 | Asian |
| USA (imported from India) | 2006 | KJ941050 | ECSA-IOL |
