# Supplementary material for: Multiple chikungunya virus introductions in Lao PDR from 2014 to 2020
Source: PLoS One. 2022 Jul 15;17(7):e0271439. doi: 10.1371/journal.pone.0271439 (PMC9286254; doi:10.1371/journal.pone.0271439)
Supplement: S3 Data — (DOCX) [file pone.0271439.s003.docx]

**Supplementary data 3.** Information relative to the Lao chikungunya samples collected between 2014 and 2020 and found positive by RT-PCR.

| Sample Identification | Date of collection | Location | Patient age (years) | Patient gender | Patient symptoms | Biological sample | Genbank accession Number |
| --- | --- | --- | --- | --- | --- | --- | --- |
| CV001 | November 2014 | Vientiane Capital (ex French Polynesia) | 53 | Male | fever, arthralgia, rash | Culture supernatant ^a^ | MZ292728 |
| 19-9293 | February 2019 | Vientiane Capital (ex Indonesia) | 44 | Male | fever, headache | Culture supernatant ^a^ | MZ292729 |
| 19-14922 | 2019 | Vientiane Capital (ex Myanmar) | 48 | Male | fever, arthralgia | Plasma | NA |
| 20-16862 | August 2020 | Vientiane Capital (ex Thailand) | 36 | Male | fever, headache, myalgia, retro-orbital pain, nausea, rash | Culture supernatant ^a^ | MZ292733 |
| 20-16656 | July 2020 | Savannakhet Province | 48 | Female | fever, arthralgia, myalgia, nausea, rash | Plasma | NA |
| 20-16657 | July 2020 | Savannakhet Province | 65 | Male | fever, arthralgia, myalgia, headache | Plasma | MZ292732 |
| 20-16926 | August 2020 | Savannakhet Province | 30 | Male | fever, headache, myalgia, retro-orbital pain, | Plasma | NA |
| 20-16927 | August 2020 | Savannakhet Province | 50 | Male | fever, headache, myalgia, retro-orbital pain, | Plasma | NA |
| 20-17063 | August 2020 | Savannakhet Province | 60 | Female | fever, headache, myalgia, retro-orbital pain, arthralgia | Plasma | NA |
| 20-17231 | August 2020 | Savannakhet Province | 68 | Female | fever, headache, myalgia, arthralgia | Plasma | NA |
| Bolikhamxay-01 | July 2020 | Bolikhamxay Province | 25 | Male | fever, arthralgia, myalgia, headache, asthenia, rash | Plasma | NA |
| Bolikhamxay-02 | July 2020 | Bolikhamxay Province | 19 | Male | fever, arthralgia, myalgia, headache, asthenia | Plasma | NA |
| Bolikhamxay-03 | 2020 | Bolikhamxay Province | 10 | Male | fever, rash | Plasma | NA |
| Bolikhamxay-05 | 2020 | Bolikhamxay Province | 24 | Female | fever, arthralgia, myalgia, headache | Plasma | NA |
| Bolikhamxay-14 | July 2020 | Bolikhamxay Province | 39 | Male | fever, arthralgia, myalgia | Plasma | MZ292730 |
| Bolikhamxay-23 | July 2020 | Bolikhamxay Province | 25 | Male | fever, rash | Plasma | NA |
| Bolikhamxay-30 | July 2020 | Bolikhamxay Province | 19 | Male | fever, arthralgia, retro-orbital pain | Plasma | MZ292731 |
| Bolikhamxay-31 | 2020 | Bolikhamxay Province | 25 | Male | fever, arthralgia, myalgia, asthenia, headache, rash | Plasma | NA |

^a^ One or two passages were performed on Vero E6 cells
